# The design of dilution loops for continuous-flow analysis

**DOI:** 10.1155/S1463924685000293

**Published:** 1985

**Authors:** Stephen C. Coverly

**Affiliations:** Technicon Instruments Company Ltd Hamilton Close Hampshire Basingstoke UK


Journal of Automatic Chemistry/*Journal of Clinical Laboratory Automation*, Vol. 7, No. 3 (Jul–Sep 1985), pp. 141–144

### The design of dilution loops for continuous-flow analysis

**Stephen C. Coverly**
*Technicon Instruments Company Ltd, Hamilton Close, Basingstoke, Hampshire, UK*

### Introduction

The sensitivity of many colorimetric and other methods of detection used in continuous-flow analysis means that samples frequently need to be diluted to bring them into the range of a particular method. On-line dilution can take the form of a dialyser, in which a proportion of the analyte diffuses through a permeable membrane into a recipient stream, or a dilution loop, where the sample is diluted, usually with water, then led back to the inlet side of the pump (hence the term 'loop'), where a small proportion is resampled. Because the resampled flow is often unsegmented, unlike the original sample flow in which the inter-sample air bubble separates the sample from the wash liquid, the dispersion in a dilution loop can be high, resulting in a lower optimum sample rate.

However, by the correct choice of fittings, and by segmenting the resample stream where appropriate, dispersion in a dilution loop can be reduced to a level where it is not the limiting factor in system performance.

### Dilution factor and flow rate

It is usually possible to achieve a given dilution factor by several combinations of flow rate. For example, a dilution of about 11 times can be effected with a sample line of 0·1 ml/min and diluent 1·0 ml/min, 0·23 sample and 2·0 diluent, 0·32 and 3·4, and so on. Considerations applying to the diluent flow rate are the stability of flow and segmentation, delay time, back pressure and diluent consumption. When a roller in a peristaltic pump lifts off the pump tube there is a momentary reduction in flow rate as the tube expands to its original shape. Thus the flow during one cycle of the pump, i.e. roller to roller, is variable, or pulsed. This effect is more marked with large diameter tubes, and the rapid variation in flow during one pump cycle can cause irregular bubble injection: for this reason tubes with a maximum flow of 2 ml/min are used when possible. When higher flow rates are necessary, the diluent can be added in two or more stages, with or without intermediate mixing, to promote regular flow at the air injection point.

It is always desirable to minimize delay time, and this corresponds to high flow rates or narrow diameter tubing. For a given diameter of transmission tubing in the lengths used in a dilution loop it is safe to pass up to eight times the flow developed by a pump tube of similar diameter. For example, the pump tube nearest in diameter to the 2 mm glass tubing used in AAII systems has a flow rate of 2.4 ml/min: thus about 10 ml/min can be pumped through a 2 mm dilution loop without developing excess back pressure, which lowers the effective pumping rate and creates problems with air injection.

The lower limit on sample flow rate is mainly determined by the size of any solids in the sample, and is normally 0·1 ml/min (0·015 in i.d.), with 0·05 ml/min (0·010 in i.d.) occasionally being used.

Thus the optimum range of dilution is up to 20 times (0.1 ml/min sample, 2 ml/min diluent), with up to about 150 times (0·05 sample, 2 × 3·9 diluent) possible. The flow rate of the resample line is generally determined by the method, and it is this flow rate which determines the choice of debubbler for unsegmented resample lines.

### Unsegmented resample lines

As with all parts of a manifold, the aim in selecting a debubbler for an unsegmented resample line is to minimize dispersion. This occurs in two parts of the resample fitting: the part connected to the resample pump tube and the part containing the segmented flow. Figure 1 shows three types of debubbler sometimes used as resample fittings. The C3 contains a ramp to remove extra or oversize bubbles – this is useful on AAI systems, but unnecessary on AAII systems with regular air injection (the intersample air bubble in a dilution system will be necessarily small). The A2 is better in this respect, but the capillary arm of both these fittings is 1·0 mm i.d., the same as that of a 0·6 ml/min pump tube. If a lower flow rate resample pump tube is used this capillary is drained slowly, creating excessive dispersion. For flow rates up to 0·6 ml/min the so-called 'modified AO' is used, with 0·025 in i.d. acidflex tubing inserted into the side arm until bubbles just pass over the top without being drawn into the resample line.

### Segmented resample lines

If a resample fitting such as the A12 or A16 is used (see figure 1), a small proportion of each bubble in the diluted stream will be drawn into the resample pump tube, segmenting the stream and thereby reducing dispersion. This reduction in dispersion can be quantified, as shown in the next section. Because the AAII system uses air injection synchronized with the pump rollers, the small resampled bubbles always occupy the same position in the resample pump tube relative to the rollers, and thus the short-term delivery is constant.

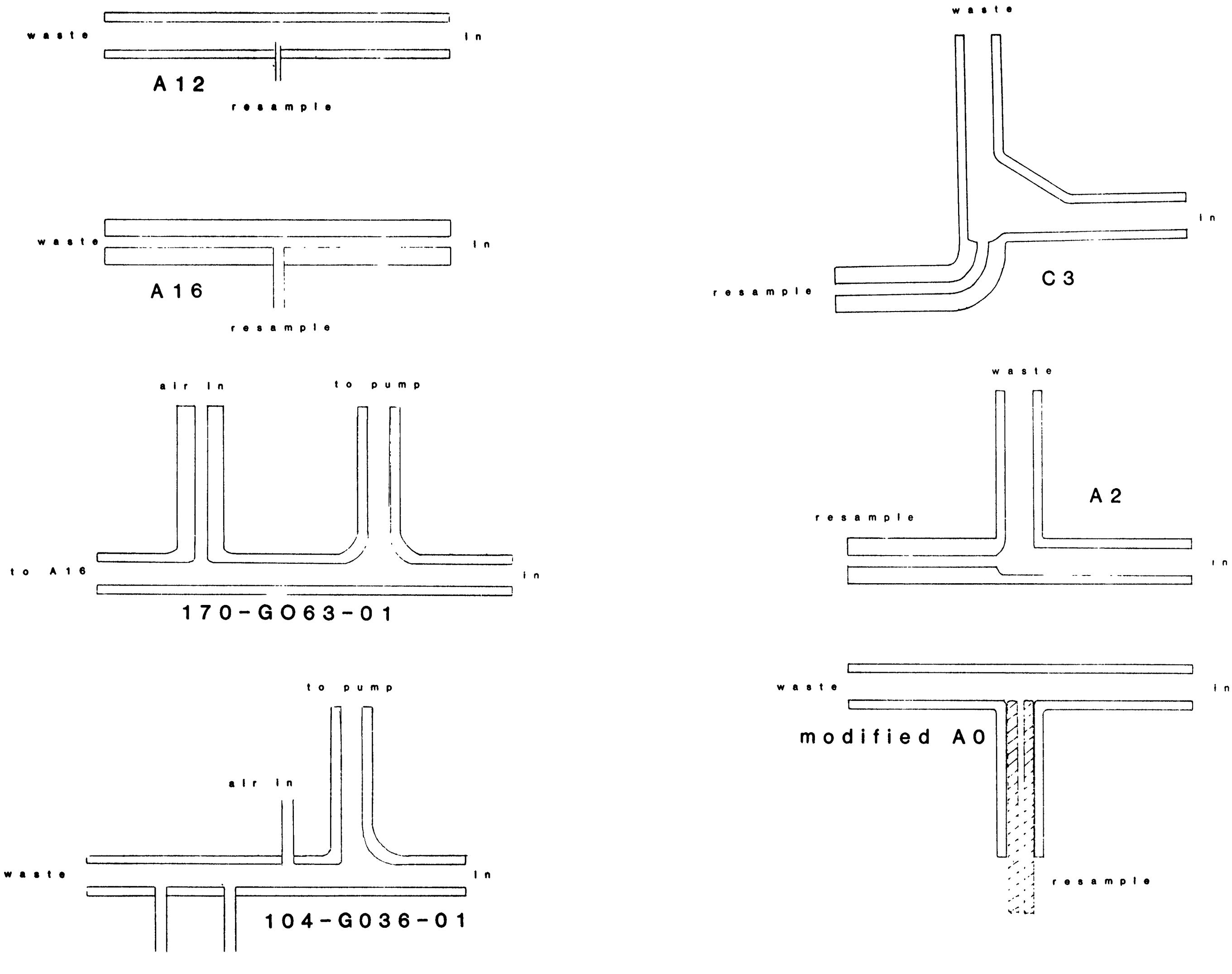

*Figure 1. Glass resample fittings for segmented and unsegmented streams.*

However, the volume of each resampled bubble depends on the velocity at which the main bubble passes across the take-off insert, and this is dependent on not only the total flow rate but also, due to the pulsed flow, on the moment in the pump cycle at which the bubble reaches the insert. The arrival time depends on the number of bubbles and liquid segments between the air injection point and the resample insert, and this is chiefly determined by the flow rate of the diluent pump tube. Because the flow rate of the tube slowly drops with age, the number of segments between these points increases, and the position which a particular bubble (for example the 60th after the injection point) occupies in the dilution loop slowly moves backwards. Thus the volume of resampled air, and therefore the delivery of liquid and the sensitivity of the method, changes with time. More serious is the short-term variation which occurs when the bubble reaches the resample insert just before or after the instant of least velocity in the cycle, as here very short variations in arrival time, such as may be caused by irregular roller spacing or intersample air bubble compression, cause large proportional changes in the resampled bubble volume, and hence a noisy response.

These problems are overcome by using a system whereby the arrival time of a bubble at the resample point does not depend on the number of segments in the loop, but only on the time within each pump cycle. This is achieved by removing the original bubble just before the resample point and immediately introducing another. The new bubble is injected in synchrony with the pump, and, because the distance between its injection point and the resample point is so short, variations in flow rate have a negligible effect on its arrival time. Part No. 170–G063–01 (see figure 1) is such a debubble/rebubble fitting, which can be used with an A12 or A16, and 104–G036–01 is a more recent type which also contains two sapphire inserts for resampling.

### Dispersion

The dispersion in a dilution loop arises from three sources: the segmented stream from the pump to the resample fitting, the resample fitting itself, and the resample pump tube.

The theoretical contribution of each can be calculated, using the equation described by Hrdina [1] which relates dispersion ($\sigma$) in seconds in unsegmented streams to diameter ($d$) in mm and time ($t$) in s:

$$4\sigma = 50\, d\sqrt{t} \qquad (1)$$

and the equation derived by Snyder and Adler [2] relating dispersion in segmented streams to flow rate ($F$) in ml/s, internal diameter ($d$) in cm, segmentation frequency ($n$) in bubbles/s, liquid viscosity ($\eta$) in poise, surface tension ($\gamma$) in dynes/cm, a mass transfer coefficient ($D$) that varies with the nature of the sample molecules and the viscosity of the liquid, and dwell time ($t$) in s.

$$\sigma^2 = \left[\frac{538\, d^{2/3}\,(F + 0{\cdot}92\, d^3 n)^{5/3}\,\eta^{7/3}}{\gamma^{2/3} F D} + 1/n\right] \left[\frac{2{\cdot}35\,(F + 0{\cdot}92\, d^3 n)^{5/3}\,\eta^{2/3}\, t}{\gamma^{2/3} F\, d^{4/3}}\right] \qquad (2)$$

A typical value for $\eta$ is 0·009, for $\gamma$ 32, and for $D$ $5 \times 10^{-5}$. Further discussions of the applications of this formula can be found in two papers by Snyder [3 and 4].

In this equation the dispersion is expressed as the variance, $\sigma^2$, of a Gaussian curve, in $s^2$.

Table 1 shows the contribution from each part of a dilution loop employing commonly-used flow rates in four configurations. Configurations ($a$) and ($b$), shown in figure 2, are both unsegmented, but ($a$) uses an A2 resample fitting and ($b$) uses a modified AO.

*Table 1. Dispersion due to different designs of dilution loop.*

| | Dispersion, $4\sigma$ (s) | | | |
|---|---|---|---|---|
| Hardware description | Segmented stream | Within fitting | Resample pump tube | Total |
| ($a$) 2 mm glassware A2 unsegmented | 7·7 | 10·1 | 19·8 | 23·5 |
| ($b$) 2 mm glassware modified AO unsegmented | 7·7 | 4·9 | 19·8 | 21·8 |
| ($c$) 2 mm glassware 170–GO63–01 + A16 segmented | 7·7 | 12·2 | 2·9 | 14·7 |
| ($d$) 1 mm glassware 104–G036–01 segmented | 5·4 | 3·8 | 2·9 | 7·2 |

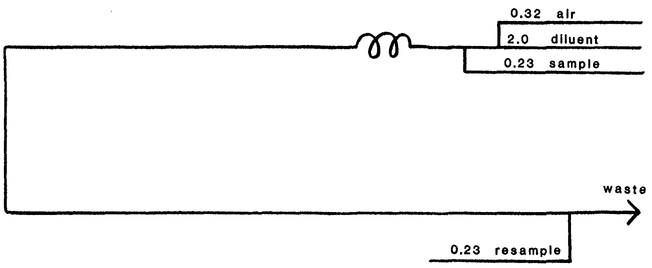

*Figure 2. Flow diagram for dilution loop with unsegmented resample stream (flow rates in ml/min).*

Configurations ($c$) and ($d$), shown in figure 3, are both resegmented, the differences lying in the resample fitting and the use of narrow bore (1 mm) tubing (Part Nos. 157–0303–03, 116–0223–16, and 116–0223–27), for the dilution loop in configuration ($d$), and conventional 2 mm glassware in configuration ($c$). The dispersions are summed using equation 3 and expressed as $4\sigma$, in s, representing the approximate sum of the rise and fall curves to 95% [1]:

$$4\sigma \text{ total} = \sqrt{4\sigma_a^2 + 4\sigma_b^2 + 4\sigma_c^2} \qquad (3)$$

These results clearly show the benefits to be obtained by the correct choice of fittings.

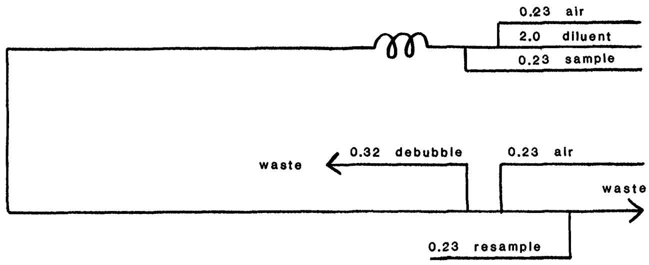

*Figure 3. Flow diagram for dilution loop with segmented resample stream (flow rates in ml/min).*

### Choice between segmented and unsegmented resample lines

It is apparent from equation (3) that the part of a system with the greatest dispersion dominates the total dispersion, and that efforts to minimize dispersion must therefore be centred here. Applying this principle to dilution loops, it is clear that the benefits of segmented resample lines are only seen when the dilution loop is one of the major contributors to system dispersion. If this is not the case it is usually preferable to use unsegmented resample lines as these are the simpler type. The figures for dispersion in table 1 can be compared to those for a 1·5 mm flowcell with a pull-through of 1·2 ml/min at 13s, a 2 mm flowcell with the same pull-through at 23s, a sample line segmented by the normal inter-sample air bubble at about 15s, and the segmented stream of a manifold incorporating a heating bath with a flow of 1·5 ml/min and a delay of 5 min at 11s.

### Comparative benefits of dilution loops and dialysers

Table 2 shows a comparison of dilution and dialysis. The principal advantages of dilution loops are their insensitivity to temperature variations and the ease of predetermining the dilution factor. As recovery in a dialyser depends on path-length, membrane type and condition, temperature, flow rate of donor and recipient streams, back pressure on both sides of the membrane, and ionic conditions, it is seldom possible to predict an exact dilution. The primary advantage of dialysis is the separation of solids and high molecular weight solutes from the analyte. The book by Furman [5] is a useful reference for this and other aspects of system design.

*Table 2. Comparison of dilution and dialysis.*

| | *Dilution loop* | *Dialyser* |
|---|---|---|
| Dilution factor | 2–150 | 3–50 |
| Temperature sensitivity | Low | High |
| Tolerance of solids | Low | High |
| Number of pump tubes | 3 or 5 | 2 or 3 |
| Approximate hardware cost | £130 | £400 |
| Dispersion, $4\sigma$ (s) | 7–25 | 12–30 |
| Lag time (s) | 55–145 | 30–80 |

### Conclusion

The design of dilution loops is governed by simple principles. Applying these principles to the requirements of a method allows systems with low dispersion and robust performance to be constructed. The theoretical dispersion of the individual parts of a system can be predicted, and this information can be used to select the most suitable dilution method for a particular application.

### References

1. Hrdina, J., *6th Colloquium on Amino Acid Analysis* (Technicon Corporation, 1967), 60.
2. Snyder, L. R. and Adler, H. J., *Analytical Chemistry* **48** (1976), 1019.
3. Snyder, L. R., *7th Technicon International Congress* (1976), 76.
4. Snyder, L. R., *Journal of Chromatography*, **125** (1976), 287.
5. Furman, W. B., *Continuous Flow Analysis* (Marcel Dekker, New York, 1976).

### ABSTRACTING

In keeping with our academic status *Journal of Automatic Chemistry incorporating Journal of Clinical Laboratory Automation* is covered by the following abstracting/indexing services:
*Current Contents/Life Sciences*
*Science Citation Index*
*ISI/BIOMED*
*Excerpta Medica*
*Analytical Abstracts*
*Chemical Abstracts*

### NOTES FOR AUTHORS

*Journal of Automatic Chemistry incorporating Journal of Clinical Laboratory Automation* covers all aspects of automation and mechanization in analytical, clinical and industrial environments. The *Journal* publishes original research papers; short communications on innovations, techniques and instrumentation, or current research in progress; reports on recent commercial developments; and meeting reports, book reviews and information on forthcoming events. All research papers are refereed.

### Manuscripts

Two copies of articles should be submitted. All articles should be typed in double spacing with ample margins, on one side of the paper only. The following items should be sent: (1) a title-page including a brief and informative title, avoiding the word 'new' and its synonyms; a full list of authors with their affiliations and full addresses; (2) an abstract of about 250 words; (3) the main text; (4) appendices (if any); (5) references; (6) tables, each table on a separate sheet and accompanied by a caption; (7) illustrations (diagrams, drawings and photographs) numbered in a single sequence from 1 upwards and with the author's name on the back of every illustration; captions to illustrations should be typed on a separate sheet. Papers are accepted for publication on condition that they have been submitted only to this *Journal*.

### References

References should be indicated in the text by numbers following the author's name, i.e. Skeggs [6]. In the reference section they should be arranged thus:

to a journal

Manks, D. P., *Journal of Automatic Chemistry*, **3** (1981), 119.

to a book

Malmstadt, H. V., in *Topics in Automatic Chemistry*, Ed. Stockwell, P. B. and Foreman, J. K. (Horwood, Chichester, 1978), p. 68.

### Illustrations

Original copies of diagrams and drawings should be supplied, and should be drawn to be suitable for reduction to the page or column width of the *Journal*, i.e. to 85 mm or 179 mm, with special attention to lettering size. Photographs may be sent as glossy prints or as negatives.

### Proofs and offprints

The principal or corresponding author will be sent proofs for checking and will receive 50 offprints free of charge. Additional offprints may be ordered on a form which accompanies the proofs.

*Manuscripts should be sent to either Dr P. B. Stockwell or Ms M. R. Stewart, see inside front cover.*

